# Regulatory T Cells in Systemic Sclerosis

**DOI:** 10.3389/fimmu.2018.02356

**Published:** 2018-10-15

**Authors:** Camelia Frantz, Cedric Auffray, Jerome Avouac, Yannick Allanore

**Affiliations:** INSERM U1016, UMR8104, Cochin Institute, Paris Descartes University, Paris, France

**Keywords:** systemic sclerosis, regulatory T cells, immune tolerance, auto-immunity, thymus

## Abstract

In recent years, accumulating evidence suggest that regulatory T cells (Tregs) are of paramount importance for the maintenance of immunological self-tolerance and immune homeostasis, even though they represent only about 5–10% of the peripheral CD4^+^ T cells in humans. Their key role is indeed supported by the spontaneous development of autoimmune diseases after Tregs depletion in mice. Moreover, there is also a growing literature that investigates possible contribution of Tregs numbers and activity in various autoimmune diseases. The contribution of Tregs in autoimmune disease has opened up a new therapeutic avenue based on restoring a healthy balance between Tregs and effector T-cells, such as Treg-based cellular transfer or low-dose IL-2 modulation. These therapies hold the promise of modulating the immune system without immunosuppression, while several issues regarding efficacy and safety need to be addressed. Systemic sclerosis (SSc) is an orphan connective tissue disease characterized by extensive immune abnormalities but also microvascular injury and fibrosis. Recently, data about the presence and function of Tregs in the pathogenesis of SSc have emerged although they remain scarce so far. First, there is a general agreement in the medical literature with regard to the decreased functional ability of circulating Tregs in SSc. Second the quantification of Tregs in patients have led to contradictory results; although the majority of the studies report reduced frequencies, there are conversely some indications suggesting that in case of disease activity circulating Tregs may increase. This paradoxical situation could be the result of a compensatory, but inefficient, amplification of Tregs in the context of inflammation. Nevertheless, these results must be tempered with regards to the heterogeneity of the studies for the phenotyping of the patients and of the most importance for Tregs definition and activity markers. Therefore, taking into account the appealing developments of Tregs roles in autoimmune diseases, together with preliminary data published in SSc, there is growing interest in deciphering Tregs in SSc, both in humans and mice models, to clarify whether the promises obtained in other autoimmune diseases may also apply to SSc.

## Regulatory T cells: gatekeepers of immunological tolerance

Human regulatory T cells (Tregs) expressing the transcription factor FoxP3 have a crucial role for the maintenance of immunological self-tolerance and immune homeostasis (1–3). The loss of dominant peripheral tolerance, exerted by Tregs, can lead to autoimmune diseases, immunopathology, allergy or metabolic disease (4, 5).

The pivotal role of Treg cells in the protection from autoimmunity is exemplified by spontaneous development of immunopathology in scurfy mice which are deficient for FoxP3^+^ Tregs (4, 6). Mutations in the human ortholog result in a similar X-linked lymphoproliferative disorder characterized by immune dysregulation, polyendocrinopathy, enteropathy, defined by the acronym IPEX (7, 8). Moreover, lack of Treg-mediated control has been shown to play a role in many animal models of autoimmunity (4) but also in numerous autoimmune disorders (9–12). In this review, we will focus on CD4^+^FoxP3^+^ Treg cell subset.

## CD4^+^ tregs: cell subsets and identification

Human Tregs were first characterized as CD4^+^CD25^+^ T cells in 2001 (13–15) based on the findings by Sakaguchi et al. that mouse Tregs constitutively express CD25, the α-chain of the IL2 receptor (16). However, CD25 is also upregulated on responder T cells upon activation. Therefore, much research has been focused on the identification of further markers to precisely distinguish the Treg population from recently activated T cells. In 2003, the transcription factor FoxP3 was shown to regulate the generation and function of Tregs in mice (17–19). Subsequently, in humans, FoxP3 was shown to be expressed predominantly by CD4^+^CD25^high^ T cells (20). However, whereas in mice FoxP3 expression seems to be restricted to Tregs (21, 22), in humans, FoxP3 is also expressed by non-regulatory CD4^+^CD25^+^ T cells (23, 24), restricting the usage of FoxP3 as a specific marker for human Tregs. Moreover, FoxP3 being an intracellular protein, it cannot be used for Tregs isolation with the goal to perform functional studies. Later, it was shown that low expression of CD127, the α-chain of the IL7 receptor, acts as an additional marker for the characterization of Treg cells among CD4^+^CD25^high^ T cells (25, 26). Indeed, FoxP3 expression and suppressive capacity are enriched in CD4^+^ T cells that express low levels of CD127. However, CD127 expression tends to be downregulated also in activated conventional CD4^+^ T cells (27, 28). Moreover, Klein et al. demonstrated that a high percentage of CD127^+^ cells expressed FoxP3 and, reciprocally, that there was a high percentage of CD127^low/−^ cells that did not express FoxP3. These results suggest that these markers did not represent the same population of Tregs (29). A number of additional cell markers for the identification of CD4^+^ Tregs have been proposed (30–32) but many of these are also induced upon activation of non-regulatory CD4^+^ T cells.

In this context, Miyara and coworkers further delineated the Treg cell compartment into three subpopulations using the combination of FoxP3 and CD45RA expression: (i) CD45RA^+^FoxP3^low^ resting Tregs (rTregs); (ii) CD45RA^−^FoxP3^high^ activated Tregs (aTregs), both of which are strongly suppressive *in vitro*; and (iii) non-suppressive cytokine-secreting CD45RA^−^FoxP3^low^ non-Tregs (33). *In vitro*, CD45RA^−^FoxP3^high^ aTregs were activated, highly suppressive and died by apoptosis after exertion of suppression, whereas rTregs were in a quiescent state, proliferated upon activation and converted into aTreg cells *in vitro* and *in vivo*. A major stake of this combination is based on the identification of a non-regulatory FoxP3^+^ T cell population, enabling to overcome the contamination by this cell population when studying Treg cells.

CD49d (α-chain of the integrin VLA-4) was also described as a marker that could discriminate contaminating effector cells from immune-suppressive Foxp3^+^ Treg cells. This marker is present on the majority of proinflammatory effector cells but absent from Foxp3^+^ Treg cells. Therefore, depletion with α-CD49d removes proinflammatory effector cells from CD25^high^CD4^+^ cells and, in combination with α-CD127, it provides access to hugely pure populations of Foxp3^+^ cells (34).

## Mechanisms of action and functional characterization of CD4^+^ treg

The best common way to analyze Treg function is based on their capacity to suppress target cell proliferation, and consist of *in vitro* suppression assays. This method relies on isolation of effector and regulatory cell populations immunomagnetically or by fluorescence activated cell sorting (FACS). Effector cells are then activated in the presence or absence of the regulatory population. After a defined period of time, their proliferation, and/or cytokine production are examined. However, FoxP3 being an intracellular protein, live human Tregs cannot be isolated using FoxP3 as a marker, and the lack of specific Treg cell surface markers precludes the isolation of a pure Treg population to test in these *in vitro* suppression assays.

Numerous mechanisms have been described as to how Tregs exert their suppressive function, including cell-cell contact dependent suppression, inhibitory cytokine release (IL-10, TGFβ, IL-35, Granzymes A et B), IL-2 deprivation, modulation of antigen-presenting cell function via CTLA-4, cytolysis and metabolic disruption of the target cell. These mechanisms have been extensively reviewed (35–38) and will not be further discussed in this article.

Defects in the number and/or function of Treg cells could each lead to a suboptimal T cell regulation, and subsequently to the development of autoimmunity.

## Systemic sclerosis

Systemic sclerosis (SSc) is an orphan connective tissue disease characterized by extensive immune abnormalities, microvascular injury and fibrosis of skin and internal organs (39). It is the most severe connective tissue disease, associated with a high mortality risk (40). Patients with SSc are classified according to skin involvement extent: limited cutaneous SSc (LcSSc), with skin involvement restricted to the hands, arms, and face; and diffuse cutaneous SSc (DcSSc), with more extensive skin thickening (truncal and proximal) and more frequent visceral involvement (41).

Although the pathogenesis of SSc is complex and remains incompletely understood (42), research in the area has confirmed that immune dysfunction is one of the most important component of the pathogenesis. Innate and adaptive immune abnormalities can be observed, and culminate in auto-antibodies production and activation of cell-mediated autoimmunity. Moreover, immune cells may trigger the complex biochemical and molecular changes that promote vasculopathy and fibrosis. Indeed, there is increasing evidence that places immune activation as a cause and not a consequence of the vasculopathy and fibrosis. First, histological studies indicate that an inflammatory infiltrate is present in the very early stages, preceding the onset of fibrosis (43). This cellular infiltrates consist mostly of T cells which are predominantly CD4^+^ cells (44). Second, fibroblasts with increased expression of type I and III procollagen mRNA can often be identified in areas adjacent to the infiltrating mononuclear cells (45, 46). Third, T cells in the skin and in the peripheral blood of SSc patients express an oligoclonal T cell receptor (TCR) repertoire, strongly suggestive of a proliferation and clonal expansion of these cells in response to a specific Ag(s) (47, 48). Furthermore, several studies have demonstrated an association of particular HLA alleles with SSc (49–52), which supports the concept of an Ag-driven T cell response in SSc. It should be noted that the genotype varies particularly strongly according to the presence of different types of autoantibodies associated with SSc: anti-centromere antibodies was associated with DRB1^*^01:01, DRB1^*^01:04, DRB1^*^01:08, DQB1^*^05:01, DPB1^*^04:02 and anti-topoisomerase I with DRB1^*^11-^*^15:02, DPB1^*^13:01 and DPB1^**^09:01 (51, 52). In a large study of HLA class II genes carried out in 1,300 SSc cases and 1,000 controls, the DRB1^*^11:04, DQA1^*^05:01 and DQB1^*^03:01 haplotypes and the *DQB1* allele were the strongest associations identified (49). The association of DRB1^*^11:04, DQA1^*^05:01 and DQB1^*^03:01 haplotype with SSc was confirmed in a similar study (944 Caucasian SSc patients and 1,320 unaffected controls) (50). Although not specific to SSc, these HLAs were not found in many other autoimmune diseases.

Therefore, among this aberrant immune response, T lymphocytes seem to be of particular importance in the pathogenesis of SSc. These cells are predominantly CD4^+^ cells, display markers of activation, with a predominant Th2 cytokine profile characterized by high levels of IL-4, IL-5, and IL-13 in skin, lung and peripheral blood (53–56). This key role of T cell proliferation and cytokine secretion in SSc suggests that this condition could be associated with a defective control of T cell activation.

## Circulating treg in SSc

Evidence for numerical and functional changes of Treg population in SSc has been obtained in several studies (Table 1). The majority of the studies reported decreased frequencies and/or impaired function of circulating Tregs in SSc patients compared to controls (12, 57–65, 74). Banica et al. investigated Treg cells in peripheral blood of patients with different connective tissue diseases, as compared with blood from healthy controls. They found a reduced percentage of CD4^+^CD25^hi^ T cells in SSc compared to controls but also to other connective tissue diseases (12). Antiga et al. also reported fewer CD4^+^CD25^bright^FoxP3^+^ cells in SSc patients naïve to any systemic treatment compared with healthy controls and with patients having other common immune-mediate dermatoses (psoriasis, atopic dermatitis) (57). This decrease was associated with reduced total TGFβ1 and IL-10 serum levels. The authors concluded that this reduced frequency of Tregs, together with that of total TGFβ1 and IL-10, may be responsible for the loss of tolerance observed in SSc. Papp and coworkers observed decreased CD4^+^CD25^+^FoxP3^+^ T cells percentages in peripheral blood of patients with SSc associated with increased Th17 cell percentages and decreased circulating IL-10 levels (59). In addition, both CD4^+^ and CD8^+^ central memory T-cell percentages were increased representing an immunologically active state. Similarly, the results of Fenoglio et al. supported an imbalanced ratio between Th17 and Treg cell subsets in SSc patients, with increased proportion of circulating Th17 cells, and decreased proportion of both CD4^+^CD25^+^CD127^−^ and CD8^+^CD28^−^ Treg cells (60). Lower frequencies of CD4^+^CD25^+^FoxP3^+^ T cells in SSc patients were reproducibly reported by other groups (62–64). Kataoka et al. found reduced frequencies of CD4^+^CD25^+^FoxP3^+^ T cells in treatment-free SSc patients compared to healthy controls, particularly in patients with late-stage disease (65).

**Table 1:** Circulating regulatory T cells in systemic sclerosis.

**Study (year) (Ref.)**	**Phenotype used to quantify**	**Study population (*n*)**	**Frequencies**	**Functionality and immune association**	**Clinical association**
**REDUCED**					
Banica et al. (12)	CD4+CD25hi	SSc: 13 Active: 7 Inactive: 6 SS: 8 SLE: 20 PM/DM: 11 HC: 18	Mean: SSc: 1.0% SS: 1.3% SLE: 1.5% PM/DM: 1.3% HC: 3.3% *p* = 0.0001 (SSc compared to HC)	NA	Treatment with glucocorticoids and immunosuppressive therapy in association was associated with reduced Tregs frequency.
Antiga et al. (57)	CD4^+^CD25^bright^FoxP3^+^	SSc^*^: 15 LcSSc: 10 DcSSc: 5 Morphea: 15 Psoriasis: 10 Atopic dermatitis: 10 HC: 10	Median (range): SSc: 1.5% (1.0–2.1) Morphea: 1.8% (1.1–2.6) Psoriasis: 2.4% (1.9–3.3) Atopic dermatitis: 2.5% (1.6–3.5) HC: 3.5 (3.1–3.9) *p* < 0.05 (SSc compared to HC)	Decreased of TGFβ1 and IL-10 serum levels	NA
Liu et al. (58)	CD4^+^FoxP3^high^CD45RA^−^ activated Treg CD4^+^FoxP3^low^CD45RA^+^ resting Treg	SSc: 31 LcSSc: 15 DcSSc: 16 HC: 33	Mean ± SD: Activated Treg SSc: 0.25 ± 0.16% HC: 0.66 ± 0.41% *p* < 0.001 Resting Treg SSc: 1.87 ± 0.94% HC: 1.63 ± 0.97% *p* = 0.320	Impaired suppressive capacity of CD4^+^CD25^+^FoxP3^+^ cells Immune imbalance Treg/Th17 with elevated Th17 cells	NA
Papp et al. (59)	CD4^+^CD25^bright^FoxP3^+^	SSc: 21 (only DcSSc) HC: 15	Mean ± SD: SSc: 5.03 ± 2.3% HC: 6.21 ± 0.1% *p* = 0.031	Suppression capability of CD4^+^CD25^+^ T cells reduced in SSc patients. Increased CD4^+^ and CD8^+^ central memory T-cell percentages. Immune imbalance Treg/Th17 with elevated Th17 cells. Decreased peripheral Il-10 level.	NA
Fenoglio et al. (60)	CD4^+^CD25^hi^CD127^low^	SSc: 36 LcSSc: 24 DcSSc: 12 HC:10	Decreased *p* < 0.05	Impaired suppressive capacity of CD4^+^CD25^hi^CD127^low^ cells in patients with diffuse and active disease. Immune imbalance Treg/Th17 with elevated Th17 cells	NA
Mathian et al. (61)	CD4^+^CD45RA^−^FoxP3^bright^CD25^bright^ activated Treg CD4^+^CD45RA^+^FoxP3^+^CD25^+^ resting Treg	SSc: 53 LcSSc: 18 DcSSc: 35 HC: 24	Median (range): Activated Treg SSc: 0.66 (0.17–2.0) HC: 1.51 (0.79–3.03) *p* < 0.0001 Resting Treg SSc: 0.72 (0.1–5.3) HC: 1.63 (0.57–4.94) *p* < 0.0001	Suppressive capacity of aTregs (CD4^+^CD45RA^−^CD25^bright^) and rTregs (CD4^+^CD45RA^+^CD25^+^): no significant difference with HC	Percentage of aTregs correlated with the EScSG activity index and the MRSS LcSSc patients had significantly less aTregs than DcSSc patients rTregs in SSc negatively correlated with disease duration Late SSc patients had significantly less circulating rTregs than early SSc patients
Cordiali-Fei et al. (62)	CD4^+^CD25^+^FoxP3^+^	SSc^*^: 25 LcSSc: 14 DcSSc: 11 HC: 15	Median (range): LcSSc: 1.67% (1.2–3.3) DcSSc: 1.85% (1.2–3.2) HC: 2.1 (1.1–3.2) *p* < 0.05	NA	Significant correlation between disease duration and reduced Treg cell percentages in LcSSc patients
Wang et al. (63)	CD4^+^CD25^+^FoxP3^+^	SSc: 18 LcSSc: 6 DcSSc: 12 HC: 17	Mean ± SD: SSc: 6.2 ± 1.8% HC: 11.1 ± 2% *p* = 0.024	Decreased FoxP3 expression in CD4+ T cells and increased methylation levels of FoxP3 gene. Inhibition of DNA methylation enhanced FoxP3 expression.	Promoter methylation status and expression level of FoxP3 significantly associated with disease activity.
Baraut et al. (64)	CD4^+^CD25^hi^FoxP3^+^	SSc^*^: 7 (only DcSSc) HC: 7	Mean ± SD: SSc: 2 ± 0.5% HC: 4.2 ± 1.1% *p* < 0.01	Impaired suppressive capacity of CD4^+^CD25^hi^CD127^low^ cells	NA
Kataoka et al. (65)	CD4^+^CD25^+^FoxP3^+^	SSc^*^: 23 LcSSc: 8 DcSSc: 15 HC: 22	Mean ± SD: SSc: 2.7 ± 1.5% HC: 4.8 ± 1.5% *p* < 0.0001	Decreased Runx1 mRNA expression in purified Treg (especially in early disease)	Reduced frequency more pronounced in late disease
**SIMILAR**					
Klein et al. (66)	CD4^+^CD25^++^FoxP3^+^	SSc: 20 LcSSc: 14 DcSSc: 6 HC: 29	Median (range): SSc: 1.1% (0.7–3.5) HC: 1.3 (0.6–2.8) *p* = 0.953	Suppressive capacity of CD4^+^CD25^+^FoxP3^+^ cells similar to that of HC	NA
Yang et al. (68)	CD4^+^CD25^+^CD127^−^	SSc: 45 LcSSc: 20 DcSSc: 25 HC: 24	Mean ± SD: Active SSc: 6.2 ± 1.2% Stable SSc: 6.5 ± 1.5% HC: 7.1 ± 1.6% *p* > 0.05	Increased Th17 cell percentages	NA
Krasimirova et al. (67)	CD4^+^CD25^+^FoxP3^+^	SSc: 24 LcSSc: 11 DcSSc: 13 HC: 16	Mean ± SD: SSc: 4.02 ± 0.52% HC: 4.16 ± 0.53%	Increased Th17 cell percentages Raised levels of IL-6, TGFβ1, IL-10, IL-17A	No association with disease activity, disease duration or visceral involvement.
**INCREASED**					
Radstake et al. (68)	CD3^+^CD25^+^FoxP3^+^CD127^−^	SSc: 68 LcSSc: 20 DcSSc: 48 HC: 26	Mean ± SEM: SSc: 17.3 ± 1.9% HC: 2.9 ± 0.5% *p* < 0.0001	Expression of CD62L and CD69 markedly lower in SSc (CD4^+^ CD25^hi^ cells) Impaired suppressive capacity of CD4^+^CD25^+^FoxP3^+^CD127^−^ cells	Significantly higher number of CD25^+^FoxP3^+^CD127^−^ cells in early DcSSc (< 2 years) compared to late DcSSc
Slobodin et al. (69)	CD4^+^CD25^bright^ FoxP3^+^	SSc: 10 LcSSc: 6 DcSSc: 4 HC: 10	Increased	Production of TGFβ1 and IL-10 by activated CD4+ cells similar in patients and controls	Correlation with the EScSG disease activity index and the Medsger disease severity index
Giovannetti et al. (70)	CD4^+^CD25^high^FoxP3^+^	SSc^*^: 35 LcSSc: 20 DcSSc: 15 HC: 39	Mean ± SD: SSc: 6.7 ± 2.2% HC: 4.5 ± 1.5% *p* < 0.0001	NA	Correlation with the EScSG disease activity index and DLCO
Rodriguez-Reyna et al. (71)	CD4^+^CD25^+^FoxP3^+^	SSc: 135 LcSSc: 78 DcSSc: 57 HC: 16	SSc: 6.0% HC: 3.3%	Increased Th17 cell percentages	NA
Jiang et al. (72)	CD4^+^CD25^+^FoxP3^+^	SSc: 53 HC: 27	SSc: 3.04% HC: 2.24% *p* = 0.018	Increased Th17 cell percentages	Association with high ILD score on CT and with low DLCO
Ugor et al. (73)	CD3^+^CD25^+^FoxP3^+^CD127^−^	SSc: 26 LcSSc: 7 DcSSc: 19 HC: 10	Increased *p* < 0.05	Increased CD62L^+^ Tregs, decreased IL-10^+^ Tregs	NA

* *Patients not receiving systemic treatment*.

*CT, computed tomography; DLCO, diffusing capacity of the lung for carbon monoxide; DcSSc, diffuse cutaneous systemic sclerosis; FC, flow cytometry; HC, healthy controls; ILD, interstitial lung disease; LcSSc, limited cutaneous systemic sclerosis; NA, not available; PM/DM, poly- and dermatomyosite; SD, standard deviation; SLE, systemic lupus erythematosus; SS, Sjögren syndrome; SSc, systemic sclerosis*.

Among other reports, some studies have reported decreased frequency of circulating Tregs but not reaching statistical significance (66, 67, 75).

Some studies have reported an increase in circulating Tregs (68–73), particularly in early phase and active disease (68–70). Radstake et al. reported an increase in the frequency of circulating CD25^+^FoxP3^+^CD127^−^ T cells in SSc patients, especially in early phase of the disease. However, despite this increase, Tregs from SSc patients harbored a defective suppressive capacity correlated with a dramatic reduction in CD62L and CD69 expression. Interestingly, co-incubation of Treg cells from healthy donors with plasma from SSc patients abrogated suppressive activity, suggesting the presence of specific soluble factors inhibiting Treg function in SSc patients (68). Two studies reported higher number of CD25^+^FoxP3^+^ T cells in SSc patients, correlated with disease activity and severity (70, 69). Three more studies reported a significantly higher frequency of circulating Tregs in SSc compared to controls, although no functional studies were performed (71–73).

These discrepancies reflect the challenge of the phenotypic characterization of Treg cells and possible contamination by activated CD25^+^ T cells. In this context, two studies have used the combination of markers described by Miyara et al. (33) discriminating CD4^+^FoxP3^low^CD45RA^+^ resting Treg (rTreg), CD4^+^FoxP3^high^CD45RA^−^ activated Treg (aTreg) from non-regulatory Foxp3^+^ cells. Mathian and coworkers found that the percentages and absolute counts for both aTreg and rTreg were decreased in SSc compared to controls, but not those for non-regulatory FoxP3^+^ CD4^+^ T cells (61). Interestingly, aTreg were decreased at any disease stage while rTreg frequency declined in late phases of SSc. Moreover, the quantitative Treg defect was less pronounced in diffuse cutaneous and/or active disease. Similarly, Liu et al. reported lower proportions of aTreg and higher proportions of non-regulatory Foxp3^+^ cells in SSc patients compared to healthy controls (58). In the latter study, the frequency of CD4^+^CD25^+^FoxP3^+^ Treg cells was significantly increased in patients with SSc, suggesting that the increase in this cell population was mainly due to elevated CD4^+^CD25^+^FoxP3^low^CD45RA^−^ non-Treg cells. These results within the same patient population support the notion that the complexity of the phenotypic characterization of this cell population explains conflicting results in the literature.

However, the discrepancies among these studies cannot be solely explained by the use of different Treg markers. Indeed, it should be emphasized that flow cytometry gating is rather subjective and depends partly on researcher selection. Therefore, studies using same Treg markers are not necessarily comparable. Moreover, the patients' characteristics, such as disease duration, disease severity and activity, concomitant treatments, might also contribute to the discrepancy among different studies as previously stated.

As regards clinical association, most of the studies reporting increased frequency of circulating Tregs have demonstrated a correlation with disease activity (69, 70) and severity (69, 72), and with early disease (68), whereas reduced frequency of Tregs seemed to be associated with late disease (61, 62, 65). Elevated Tregs were also reported in patients with a high interstitial lung disease (ILD) score on computed tomography (72) and with low DLCO (70, 72). No other clinical association was found, in particular, no difference was observed between the two subsets of the disease.

Effect of medications on Tregs frequency have been raised by some authors. It is of note that most of the patients in studies reporting increased or similar Tregs frequency received systemic treatment which may have bias the results, since some authors reported that immunosuppressive therapy is able to increase the pool of circulating Treg (76, 77). On the other hand, immunosuppressive therapy and bosentan showed no significant effect on the frequency of Tregs in some reports (66, 73). In contrast, treatment with glucocorticoids and immunosuppressive therapy in association was associated with reduced Tregs frequency in the study of Banica et al. (12). Thus, more investigations are needed to evaluate the impact of therapies on Treg cells.

Regarding the functional capacity of circulating Tregs, almost all studies agree that Tregs fail to produce inhibitory cytokines or suppress the effector T cells in SSc (58–60, 64, 68).

Pulmonary arterial hypertension (PAH) is one of the most severe complication of SSc. Several studies have investigated the role of Tregs in PAH. Two studies reported elevated CD4^+^CD25^+^FoxP3^+^ T cells in the peripheral blood of idiopathic PAH patients (78, 79), but not in the lungs (78). Huertas et al. investigated the functional status of CD4^+^CD25^+^FoxP3^+^ Treg function by measuring Treg STAT3 phosphorylation in patients with idiopathic PAH, heritable PAH or SSc-PAH, compared to controls (80). Although Treg cell numbers were similar between patients and controls, they found that Tregs were dysfunctional in all these PAH subgroups, including SSc-PAH, with reduced proportion of Treg-pSTAT3^+^ cells compared to controls.

## Mechanisms of tregs dysfunction in SSc

Mechanisms of Tregs dysfunction in SSc have been investigated by several studies. Aberrant epigenetic modifications, such as microRNA, DNA methylation, histone modifications, affecting FoxP3 and other key genes in Tregs have been shown to contribute to disease activity and tissue inflammation in autoimmune diseases (81). In SSc, Wang et al. reported elevated methylation levels of the FoxP3 promoter, inversely correlated with FoxP3 mRNA expression, and accompanied by reduced proportion of CD4^+^CD25^+^FoxP3^+^ Tregs (63). Furthermore, treatment of SSc CD4^+^ T cells with 5-azacytidine, a DNA methylation inhibitor, reduced the mean methylation levels, increased FoxP3 expression and induced Treg generation. Interestingly, the promoter methylation status and expression level of Foxp3 were significantly associated with disease activity. D'Amico et al. provide evidence of the association between rs2294020 FoxP3 polymorphism and disease progression in a female Italian population (82). Otherwise, it has been reported a significantly higher frequency of skewed X chromosomal inactivation in patients with SSc compared with controls, correlated with lower FoxP3 expression in CD4^+^CD25^+^ cells and less efficient suppressive activity (83). Kataoka and al. reported reduced expression of the transcription factor Runx1 mRNA correlated with decreased proportion of Tregs even in early stages of the disease (65). Semaphorin 3A serum levels along with cell expression on Tregs were reported to be low in SSc patients (84).

In addition, recent evidence indicate that Tregs could contribute to SSc pathogenesis by conversion into pathogenic effector T cells in the presence of appropriate environment. Thus, because accumulating evidence suggest that Th17 cells could be responsible for prominent features of SSc (53, 71, 85), it has been hypothesized that a Treg/Th17 imbalance could be a pivotal component of SSc pathogenesis. Indeed, Fenoglio et al. found a significant correlation between increased circulating Th17 cells and alteration of the Treg compartment (60). One could argue that the observed decrease in Treg cells could be the result of conversion to Th17. Several groups have reported the conversion of Tregs to Th17 cells in both mouse and human (86–88), supporting this hypothesis. Moreover, IL-6 and IL-1β, that are highly expressed in inflammatory conditions, have been shown to convert Tregs to Th17 cells (89, 90). Liu et al. found that CD4^+^CD25^+^FoxP3^low^CD45RA^−^ non-regulatory T cells produced high levels of IL-17 (58). They hypothesized that this population of FoxP3^+^ non-regulatory T cells expressing IL-17 could represent a transitional phase in the conversion process from Treg to Th17 cells. Consistent with this result, T cells that co-express IL-17 and FoxP3 have been identified by other groups (91, 92). In contrast, although they reported decreased Tregs proportions, no IL-17 amplification was observed in blood and skin of SSc patients in the study of Mathian et al. (61).

More recently, MacDonald et al., using flow cytometry, analyzed FoxP3 and cytokine expression among skin-resident T cells isolated from cultured explants (93). They found that Tregs from SSc-skin produced significant amount of Th2 cell-associated cytokines IL-4 and IL-13 compared to controls. On the other hand, circulating Tregs of SSc patients did not produce Th2 cytokines, but they contained a significantly higher proportion of skin-homing cells expressing Th2 cell-associated chemokine receptors. The authors also found evidence that IL-33 might be an important stimulator of tissue-localized loss of normal Tregs function and polarization into Th2-like cells. Altogether, these results further support the hypothesis that the skin of SSc patients provides the appropriate environment for transdifferentiation of Tregs toward a Th2-like phenotype, that might contribute to fibrosis in patients with SSc.

## Tregs at the site of inflammation in SSc

When studying Treg cells in such diseases, one needs to consider potential differences between Treg cells derived from the peripheral blood vs. the inflamed organs (skin, lungs) in terms of function and frequency. In contrast to the lung, skin-resident Tregs are being actively investigated, probably due to relative ease of access for tissue samples. Therefore, data from mice and human subjects have revealed the importance of correct Treg cell positioning in the skin for the maintenance of immune homeostasis and prevention of spontaneous autoimmune and inflammatory disease (94). Contradictory results have been reported by the few studies investigating the presence of Tregs in the skin of patients with SSc (Table 2). Earlier studies have found fewer FoxP3^+^ cells by immunohistochemistry as compared to healthy controls or control diseases (psoriasis and atopic dermatitis) (57, 66). Interestingly, no significant difference was revealed when comparing lesional and non-lesional skin of SSc patients (66). This decrease was associated with reduced TGFβ and IL-10, which are regulatory cytokines involved in Treg suppressive function, both in skin and blood of SSc patients in the study of Antiga and coworkers (57). By contrast, in the study of Yang et al. FoxP3^+^ Treg cells was reported to be enriched in both the dermis and epidermis of patients with early SSc compared with patients with late SSc and healthy controls (75). The authors hypothesized that this expansion of FoxP3^+^ cells in early SSc skin may reflect a regulatory feedback mechanism to restore cellular tolerance and ameliorate harmful autoimmune responses. It should be noted that disease duration was not reported in the two studies that have found fewer FoxP3^+^ cells. More recently, MacDonald et al., found that FoxP3^+^ cells with high IL-4 and IL-13 production could be detected more frequently in the skin of SSc patients compared to normal controls (93). This study provides the first evidence for the differentiation of human Treg cells into Th2 cytokine-producing cells that might contribute to fibrosis in patients with SSc.

**Table 2:** Regulatory T cells in systemic sclerosis skin.

**Study (year) (Ref.)**	**Phenotyped by**	**Phenotype used to quantify**	**Study population**	**Frequencies**	**Functionality and immune association**
**INCREASED**					
Yang et al. (75)	IHC	FoxP3^+^ cells	SSc: 13 LcSSc: 1 DcSSc: 12 HC: 4	Mean ± SD: Early SSc: Superficial dermis: 10.5 ± 1.6%; deep dermis: 6.9 ± 1.7% Late SSc: Superficial dermis: 2.2 ± 1.3%; deep dermis: 1.2 ± 10.8% HC: Superficial dermis: 0.8 ± 0.4%; deep dermis: 0.8 ± 0.4% *p* < 0.01	Higher in early disease
MacDonald et al. (93)	FC	FoxP3^+^ cells	SSc: 19 HC: 13	Mean ± SD: SSc: 30.3 ± 2.8% HC: 23.7 ± 4.5% *p* > 0.05	Production of high amounts of Th2 cell-associated cytokines IL-4 and IL-13 by Tregs from skin
**DECREASED**					
Antiga et al. (57)	IHC	FoxP3^+^ cells	SSc^*^: 15 LcSSc: 10 DcSSc: 5 HC: 10	Median (range): SSc: 2% (1–4.5) HC: 9% (4.2–10)	Reduced TGFβ^+^ and Il-10^+^ cells
Klein et al. (66)	IHC	FoxP3^+^ cells among CD4^+^ cells	SSc: 12 Psoriasis: 10 Lichen planus: 10 Atopic dermatitis: 10	Median (range): SSc: 17.2% (9.1–21.7) Psoriasis: 45.4% (14.9–57.7) Lichen planus: 51.3 (16.3–78.7) Atopic dermatitis: 33% (17–55.1) *p* < 0.005 (compared with control diseases) No significant difference when comparing lesional and non-lesional skin biopsies of SSc patients	NA

* *Patients not receiving systemic treatment*.

*DcSSc, diffuse cutaneous systemic sclerosis; FC, flow cytometry; HC, healthy controls; IHC, immunohistochemistry; LcSSc, limited cutaneous systemic sclerosis*.

## Tregs in SSc mouse models

Numerous inducible and genetic mouse models of SSc have been developed and characterized in the last years (95). Conversely to other autoimmune diseases, very scarce data about Tregs in SSc mouse models have been produced. In the topoisomerase mouse model, treatment with topoisomerase I and Freund's complete adjuvant (CFA) induces SSc-like skin, lung fibrosis and autoimmune abnormalities with anti-topoisomerase I auto-antibody production (96). This was associated with increased IL-6, TGFβ1, and IL-17 production and decreased IL-10 production. In this model, mice treated with topoisomerase I and CFA exhibited significantly increased frequencies of Th1 cells, Th2 cells, Th17 cells and Treg cells in bronchoalveolar lavage fluid compared with mice treated with saline or with topoisomerase I. Functional characteristics of Tregs was not assessed in this study.

In the mouse model of bleomycin-induced pulmonary fibrosis, contradictory results have been produced. Birjandi et al. found that treatment by IL-2 complex, used to expand CD4^+^CD25^high^FoxP3^+^ cells in the lung, leads to immune deviation that is dominated by type 2 immune response within the lung, and associated with exacerbate lung fibrosis (97). Moreover, they showed that bleomycin had a modifying and profibrotic effect on the CD4^+^CD25^high^Foxp3^+^ cells. This was corroborated by adoptive transfer experiments in Rag^−/−^ mice. The authors concluded that a therapeutic strategy of expanding CD4^+^CD25^high^FoxP3^+^ in humans may be harmful via the augmentation of Th2 immune responses in patients with idiopathic pulmonary fibrosis and other fibroproliferative lung diseases. On the other hand, adoptive transfer of Tregs on day 14 after a bleomycin challenge significantly reduced pulmonary fibrosis in another report (98). Moreover, although splenocytes significantly improved bleomycin-induced pulmonary fibrosis when they were administered on day 14, this effect was abolished by depleting Tregs with an anti-CD25 monoclonal antibody. Finally, another group found that early depletion of Tregs with an anti-CD25 antibody led to favorable outcomes whereas late depletion of Tregs led to increased fibrosis, suggesting that Tregs play a detrimental role in early stages but protective role in late stages of pulmonary fibrosis in mice (99).

Although these results have to be taken into account in future therapeutic strategy using Treg cells in such diseases, it should be noted that extrapolation of these data from a mouse model to human is challenging since bleomycin may not accurately recapitulate human SSc ILD or other human fibrotic lung diseases. Moreover, there remains much controversy in the field about the actual role of Tregs.

## Therapeutics application

Taking into account that Tregs are immunodominant suppressors, there is a huge interest in the therapeutic potential of Tregs in several immune-mediated diseases. Indeed, adoptive cellular therapies may offer fewer risks and better efficacy than traditional pharmacological strategies. So far, clinical research has involved mostly hematopoietic stem cell transplantations, solid organ transplantations, and autoimmunity. Mechanisms of actions are incompletely understood but Tregs protect from auto-aggression and damage to tissues; the effect is executed mainly via cell-to-cell contacts, and also “control by starvation/theft” of IL-2. In general, what is critical is the balance between Tregs and effector T cells (Teff). Therefore, changing the balance between Tregs and Teff is a promising avenue to restore immune homeostasis and to treat autoimmune diseases. Moreover, since the recognition of antigen is a central part in Treg function and their therapeutic use, the modulation of T cell receptor specificity may be offer very stimulating perspectives (100).

Early reports demonstrated that adoptive transfer of Treg cells associated with hematopoietic stem cell transplantation (HSCT) in mice promoted the graft vs. leukemia effect (GvL) and protected from graft vs. host disease (GvHD). Unfortunately, this simple strategy cannot be translated to humans, such as the clinical efficacy requires the administration of a high number of cells whereas Treg cells represent a very low percentage of leukocytes in the blood. Thus, manufacturing procedures to expand Tregs *in vitro* before administration was developed. Multiple sclerosis (MS) is a well-defined autoimmune disease with solid evidence of Treg involvement. Remarkably, remission or prevention of experimental autoimmune encephalomyelitis (EAE), was associated with the induction of CD4^+^CD25^+^ Tregs. The adoptive transfer of Tregs further confirmed this hypothesis: transfer before EAE induction prevented EAE, and transfer to mice that already had EAE alleviated symptoms (101).

In humans, infusion of Tregs that is the direct approach to increase Tregs have been used in several phase 1 clinical trials for the prevention of GvHD or type 1 diabetes. Currently, several trials are registered in clinicaltrials.gov website for various conditions, such as liver transplantation (NCT01624077), autoimmune hepatitis (NCT02704338), chronic graft vs. host disease (NCT02385019 and NCT01937468), kidney transplantation (NCT02088931), type 1 diabetes (NCT01210664), systemic lupus erythematosus (NCT02428309). Although it is manageable to produce large numbers of alloantigen-reactive Tregs through selective stimulation by allogeneic antigen presenting cells, producing a high number of tissue antigen-specific Tregs for autoimmune diseases is far more challenging. Indeed, the low frequency of Treg precursor and the tendency of Tregs to destabilize after repeated *in vitro* stimulation impair easy large quantity production (102, 103).

Manipulating Tregs is another avenue that may even be complementary to adoptive cell transfer. In order to restore a safe equilibrium between Treg and Teff, anti-CD3 strategies mainly using antibodies was first developed (104, 105). However, efficacy was restricted to some patients and only at early stages. Co-stimulation may also be used and low-dose CTLA4-Ig therapy can enhance Treg and prevent immune activation (105). Finally, the most stimulating findings have been reported with IL2 and relate to the harboring by Tregs of high affinity IL-2 receptor promoting preferential expansion in conditions of low amounts of IL-2. Very stimulating data have been reported in humans in type 1 diabetes (106), GvHD (107), and regrowth of the scalp and/or body hair could be seen in alopecia areata (108). Patients with hepatitis C virus-induced vasculitis have a set of symptoms including fatigue, skin purpura, arthralgia, neuropathy and kidney involvement. In eight out of ten patients treated with low-dose IL-2, these symptoms progressively disappeared. It must be pointed out that in most cases, clinical improvements started to be observed after the second or third course of IL-2 therapy (109). Systemic lupus erythematosus (SLE) shares with SSc several immune disturbances. A dysbalance between Treg and Teff was recently confirmed in SLE with correlations between these findings and disease activity. *In vitro* experiments showed that lack of IL-2 production by CD4^+^ T cells accounted for the loss of CD25 expression in SLE Treg (110). Preliminary data in few patients receiving low-dose IL2 showed effectiveness to expand Treg (111). Clinical effects are now under investigations. Thus, IL-2 therapy is a promising avenue for expanding Treg cells and improving clinical outcomes for patients with autoimmune disease and trials are ongoing in connective tissues diseases including systemic sclerosis (TRANSREG study NCT01988506).

Similarly, anti-CD25 therapy results in prevention of activation and proliferation of T cells and inhibition of T cell responses. It is indicated for the prevention of acute organ rejection in adult and pediatric renal transplant recipients in combination with other immunosuppressive agents and has been studied in some immune diseases. Basiliximab is a chimeric (human/murine) anti-CD25 monoclonal antibody. It was administered to 10 SSc patients with severe skin involvement in addition to concomitant immunosuppressive and vasoactive treatments (112). Outcomes showed a reduction in skin fibrosis at week 68 and improvement in lung function at week 44. Treatment with basiliximab was well-tolerated. Although erythema, transient nausea, fatigue and weakness were common, severe reactions with significant dyspnea occurred in only one case. No patient had a documented severe infection and only one patient needed antibacterial therapy because of suspected respiratory infection. However, the application of this therapy in auto-immune diseases could be questionable. Indeed, since CD25 is also expressed on regulatory T cells, elimination or inhibition of the functional capacity of this subset using CD25 antibody might be counterproductive.

Use of autologous hematopoietic stem cell transplantation SCT (aHSCT) has recently gained interest in systemic sclerosis (64, 113–115) although the right regimen and best patient profile remain a matter of debate. It must be pointed out that sustained regression of skin and lung fibrosis has been reported for some patients. After aHSCT, reappearance of functional B cells, T-cell development, reconstitution of effector cells and efficient antigen presentation to reconstitute the pre-transplantation immune repertoire has been described. In SSc, there is scarce data about Treg restoration after aHSCT and contradictory results have been reported. A preliminary report about 7 patients focused on Treg showing a decrease number of Treg at baseline and altered suppressive capacity contrasting with restoration of Treg numbers and suppressive activity 24 months after aHSCT although a high variability was observed (64). On the other hand, in another report about 11 patients, both CD4^+^ activated effector T cells and Tregs did not reconstitute well after aHSCT with high dose cyclophosphamide conditioning, although the patients displayed resolution of clinical SSc (115). Therefore, the authors concluded that a complete reconstitution of the immune system, including Tregs, is not necessary for a treatment effect. Besides reconstitution of Treg numbers and suppressive capacity, increase in diversity of the TCR repertoire of Treg could be a crucial mechanism for the re-establishment of immune tolerance after aHSCT (116). Thus, future research into the effect of aHSCT on Treg cell compartment in SSc is required to clarify the underlying mechanisms of Treg cell pool renewal and the potential link with clinical outcome.

## Conclusion

Evidence for numerical and functional changes of Tregs in SSc has been obtained in several studies. While the majority of the studies reported reduced frequencies of circulating Tregs in SSc patients compared to controls, it seems that some patients, especially in early phase and active disease have increased number of circulating Tregs. This paradoxical situation could be the result of a compensatory, but inefficient, amplification of Tregs in the context of active inflammation. In addition to diminished suppressive capacity, recent evidence indicate that Tregs could contribute to SSc pathogenesis by conversion into pathogenic effector T cells in the presence of appropriate environment, such as Th17 cells and Th2 cytokine-producing cells.

Nevertheless, these results should be tempered with regards to the heterogeneity of the studies in terms of patient's phenotype, and of the most importance regarding Tregs definition and activity markers. Moreover, although most previous studies analyzed peripheral blood of patients, the studies on Treg cells investigating phenotype and function in the site of inflammation are still sparse. Furthermore, conversely to other auto-immune diseases, very scarce data about Tregs in SSc mouse models have been produced. Thus, since the available data points toward a central role of Treg cells in SSc, future research is definitively needed to clarify the role of this cell population in SSc pathogenesis.

Finally, since the effect of existing treatment modalities on Tregs in SSc has not been elaborated sufficiently, gaining a better understanding of the natural history of Treg function and the affected mechanisms in SSc will certainly lead to new avenues in therapy, and will help to clarify whether the promises obtained in other autoimmune diseases may also apply to SSc.

## Author contributions

CF wrote the first draft of the manuscript; YA wrote sections of the manuscript. All authors contributed to manuscript revision, read and approved the submitted version.

### Conflict of interest statement

The authors declare that the research was conducted in the absence of any commercial or financial relationships that could be construed as a potential conflict of interest. The reviewer SS and handling Editor declared their shared affiliation.
